# Wheat Seedling Emergence from Deep Planting Depths and Its Relationship with Coleoptile Length

**DOI:** 10.1371/journal.pone.0073314

**Published:** 2013-09-03

**Authors:** Amita Mohan, William F. Schillinger, Kulvinder S. Gill

**Affiliations:** Department of Crop and Soil Sciences, Washington State University, Pullman, Washington, United States of America; United States Department of Agriculture, United States of America

## Abstract

Successful stand establishment is prerequisite for optimum crop yields. In some low-precipitation zones, wheat (*Triticum aestivum* L.) is planted as deep as 200 mm below the soil surface to reach adequate soil moisture for germination. To better understand the relationship of coleoptile length and other seed characteristics with emergence from deep planting (EDP), we evaluated 662 wheat cultivars grown around the world since the beginning of the 20^th^ century. Coleoptile length of collection entries ranged from 34 to 114 mm. A specialized field EDP test showed dramatic emergence differences among cultivars ranging from 0–66% by 21 days after planting (DAP). Less than 1% of entries had any seedlings emerged by 7 DAP and 43% on day 8. A wide range of EDP within each coleoptile length class suggests the involvement of genes other than those controlling coleoptile length. Emergence was correlated with coleoptile length, but some lines with short coleoptiles ranked among the top emergers. Coleoptiles longer than 90 mm showed no advantage for EDP and may even have a negative effect. Overall, coleoptile length accounted for only 28% of the variability in emergence among entries; much lower than the 60% or greater reported in previous studies. Seed weight had little correlation with EDP. Results show that EDP is largely controlled by yet poorly understood mechanisms other than coleoptile length.

## Introduction

Deep planting of wheat into stored soil moisture is practiced in Mediterranean-like climate regions, including the Pacific Northwest (PNW) of the United States, parts of western and southwestern Australia, central Chile, and several countries surrounding the Mediterranean Sea. Winter wheat in the low-precipitation (<300 mm annual) region of the PNW is planted 100 to 200 mm below the surface of summer-fallowed soils with deep-furrow drills to reach adequate moisture for seedling emergence [1]. Introduction of high-yielding semi-dwarf wheat cultivars in the early 1960s spawned the “green revolution”. Height reduction in semi-dwarf cultivars was due to mutations in *Rht-B1* (*Rht1*) or *Rht-D1* (*Rht2*) genes that reduce either the production or the perception of gibberellin (GA), an important plant growth hormone [2]. Semi-dwarf wheat cultivars are lodging resistant, thus allowing application of higher fertilizer inputs, and have improved harvest index compared to standard-height cultivars due to increased partitioning of assimilates to reproductive organs resulting in more fertile florets per spikelet [3]–[5]. However, semi-dwarf cultivars are not popular among farmers in some low-precipitation Mediterranean regions because of their relatively poor and/or slow emergence from deep planting depths [6]–[8]. Decreased response to endogenous GA in *Rht-B1b* and/or *Rht-D1b gene* (GA-insensitive) mutants results in reduced cell size and elongation with corresponding reduction in coleoptile length, plant height, and leaf area [9]–[13]. Compared to GA-insensitive mutants, GA-responsive mutants (*Rht8*) are reported to reduce plant height without reducing coleoptile length [9], [14] and thus have less negative effect on emergence.

Wheat seedling emergence does not have linear relationship with coleoptile length as many other factors have been implicated in the process. Emergence and coleoptile length is reported to be influenced both by genetic background and environmental factors including soil texture, seed-zone water content, temperature, light penetration, and crop residue [15]–[20]. Increased seed size has been reported to positively affect early seedling vigor in many crop species [21]–[23], although about an equal number of reports argue against this correlation [18], [24]. Similarly, reports are mixed on the effect of seed weight on coleoptile length of wheat, with no effect reported for semi-dwarfs but a positive effect on some tall durum lines [25].

The pleiotropic effects associated with GA insensitive mutants on early growth have been reported and coleoptile length was positively correlated with plant height at maturity [6], [8], [26]. The major phenotypic effect quantitative trait loci (QTL) for coleoptile length were mapped on the short arm of 4B and 4D chromosomes in the close proximity of dwarfing genes, thus accounting for the pleotropic effect of dwarfing genes on coleoptile length [27]. Moreover, small-effect QTLs with additive effects were identified on wheat chromosomes 1A, 2B, 2D, 3B, 3A, 5A, 6A [27], [28]. Further, genetic background of the cultivar and environmental conditions influence the relation between coleoptile length with plant height. This suggests the possibility to select for a longer coleoptile in reduced plant height genotypes [6], but more detailed systematic study is needed to understand the mechanism behind the relationship of coleoptile length, emergence, and plant height.

Our study was undertaken to gain a better understanding of wheat seedling emergence from deep planting depths and its relation to coleoptile length along with plant height, seed weight, and size. Another objective of the study was to identify the fastest and best emerging wheat cultivar to serve as a donor to improve the emergence trait in new and existing PNW cultivars. We used a population of 662 wheat cultivars from the major wheat growing areas of the world to capture variation present among cultivars. The collection included both winter and spring type habit and represented all major market classes of wheat.

## Materials and Methods

### Ethics Statement

Field experiments were conducted at the Washington State University (WSU) Dryland Research Station located near Lind, WA. No special permissions were required to conduct the experiment. The field studies did not involve any endangered or protected species. The wheat germplasm used in the study was obtained from the Germplasm Resources Information Network (GRIN), CIMMYT, and from WSU historical collection. These are open access seed sources available for research and education purposes.

### Germplasm

We collected 662 wheat cultivars grown around the world; 360 with winter and 302 with spring growth habit (Table S1). The collection included 96 cultivars from the PNW, 125 from a historical US wheat collection including cultivars released since 1871, and 80 from the International Maize and Wheat Improvement Center (CIMMYT), Mexico representing the major wheat growing regions of the world. The remaining 361 entries were US cultivars collected from major wheat growing regions (outside the PNW) that have been widely grown since 1950. The most popular cultivars during each 5-year interval since 1950 from all major breeding programs in the US were included. Our intention was to capture as much variation among cultivated wheat as possible without making the population size unmanageably large. The world collection also represented different market classes depending upon hardness, color, and kernel shape. The collection consisted of 112 soft white winter (SWW), 90 soft white spring (SWS), 101 soft red winter (SRW), 35 soft red spring (SRS), 97 hard red winter (HRW), 127 hard red spring (HRS), 9 hard white winter (HWW), 57 hard white spring (HWS), and 34 club wheat cultivars. Due to concerns about seed purity and vigor, a single seed of each entry was grown in the greenhouse to obtain single-plant seeds. The harvested single-plant seed was then multiplied in the greenhouse and used for all the experiments.

### Coleoptile Length Measurement

For coleoptile length measurement, 15 uniform-sized seeds of each cultivar with no physical damage were placed in the middle of a moist germination paper (Heavy Germination paper #SD 7615L), about one centimeter apart with germ end down. The germination paper was then folded vertically in half with the seed placed in the crease, the folded half was again folded horizontally four times and placed in a plastic tray with holes at the base to drain excess water. The plastic trays were then placed inside a completely darkened box and kept in a growth chamber at a constant temperature of 22°C. After 10 days, the average coleoptile length of 10 randomly-selected seedlings was recorded to the nearest millimeter measuring from the base of the seed to the coleoptile tip. Germination percentage of all the lines was recorded.

### Field Test for Emergence from Deep Planting

Emergence from deep planting was conducted at the WSU Dryland Research Station near Lind, WA during the 2009–2010 crop season. The experiment was planted on land that had been fallowed for 13 months (i.e., since the last wheat harvest). Soil type was Shano silt loam (coarse-silty, mixed, superactive, mesic Xeric Haplocambids) with a soil textural size distribution of 10% clay, 51% silt, and 39% fine sand. Slope was <2%. Soil volumetric water content in the seed zone was determined gravimetrically at time of planting on September 3. These measurements were obtained in 2-cm increments avoiding wheel tracks, to a depth of 24 cm using an incremental soil sampler. Mean water content for each increment was determined from four soil cores.

For each entry, 50 uniform-sized seeds with no physical damage were planted using a four-opener deep-furrow drill in 3-m-long rows with a 0.38-m spacing between rows. The experimental design was randomized complete block with four replicates per entry. To account for any differences among openers, each entry was planted once with each of the four openers.

Seeds were planted 150 mm below the soil surface into a seed zone having a water potential of −0.50 MPa (11.5% water by volume). The depth of the soil covering the seeds was 125 mm. Wheat seedling emergence was determined by counting individual seedlings at 24-hour intervals. Seedling emergence counts were obtained on 6, 7, 8, 9, 10, 15, and 21 DAP. No rainfall occurred at the site from time of planting through 21 DAP.

### Plant Height, Seed Weight and Seed Size

Plant height of each entry was recorded at maturity by measuring the average distance from the soil surface to the top of the spike (excluding awns) of the three tallest plants. Several entries (mainly spring types) were winterkilled and, therefore, plant height could obviously not be obtained. Thousand kernel weight (TKW) for each entry was determined using electronic counter (Oldmill Company, Model 850.2) and then weighing on a digital scale. Seed size and grain hardness was determined using 200 seeds of each entry with the Single-Kernel Characterization System (Perten Instruments, Springfield, IL, Model 4100).

### Statistical Analysis

Data analysis was conducted using the PROC GLM procedure of Statistical Analysis System (SAS; SAS Inst. 2000). Means and standard deviations were determined using PROC MEANS in SAS. Analysis of variance was performed using PROC MIXED (SAS Institute, 2000). Pearson’s linear correlation coefficients were calculated by PROC CORR. Significance level was tested at P<0.05. Graphical representation of data was conducted using JMP genomics (JMP, Version 10. SAS Institute Inc., Cary, NC, 1989–2007).

## Results

### Coleoptile Length Variation

**Figure 1:**
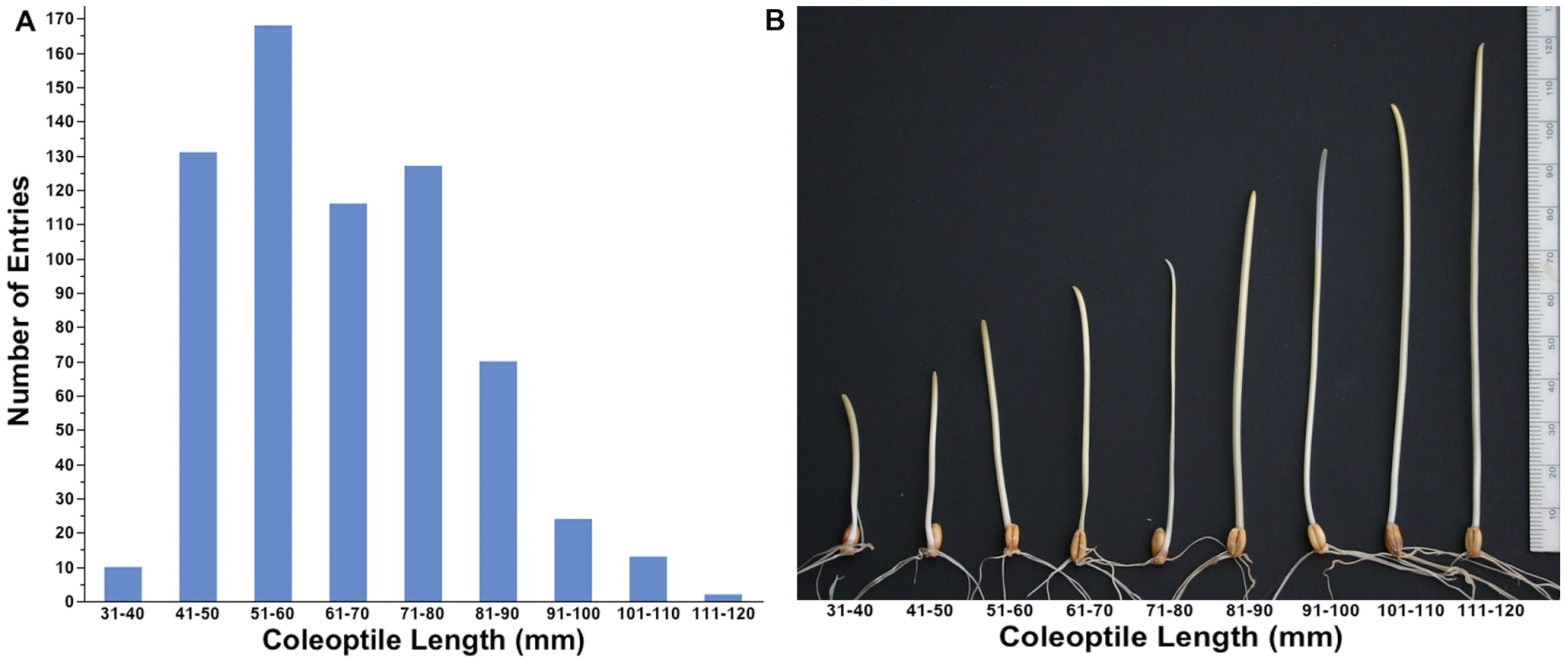
Coleoptile length distribution among 662 entries of the world wheat collection in 10-mm intervals (A). Representative picture of coleoptiles in each 10-mm increment class interval (B).

Mean coleoptile length among entries ranged from 34 mm to 114 mm (Figure 1A–B). The vast majority of entries had a mean coleoptile length between 41 to 90 mm. Only 10 of the 662 lines had coleoptiles shorter than 41 mm and 40 lines had coleoptiles longer than 90 mm (Figure 1A). The median coleoptile length was 62.4 mm. Among the nine coleoptile length intervals made in 10 mm increments, the interval 51 to 60 mm accounted for the largest number of entries (168). Coleoptile length of spring wheat lines ranged from 37 to 114 mm with a median of 62 compared to the winter lines with a range of 34 to 103 mm and a median of 63. Among various grain market classes, mean coleoptile length was the highest (71) for club wheat and the lowest for white classes (HWW, HWS, SWW), having a mean coleoptile length of 60 mm. The mean coleoptile length was 68 mm for SWS, 66 mm for both SRW and HRW, 65 mm for SRS and 64 mm for the HRS classes.

### Variation in Emergence from Deep Planting Depth

The fastest emergence was recorded in four cultivars (Spinkcota, Regent, Pacific Bluestem, ARS95-452) that had an average of at least two emerged seedlings on 7 DAP. By 8 DAP, 43% of the entries had an average of at least one emerged seedling, and this was 96% on 9 DAP. No differences were observed for EDP between spring and winter growth habit. By 21 DAP, 658 lines (99.4%) had some emergence while four entries (0.6%) did not emerge at all. Of the entries that emerged, the extent of emergence of total seeds planted ranged from 4% to 66% by 21 DAP. Emergence in some lines was fast and uniform (e.g., Spinkcota, Indian, White Federation, and Barbee) and was completed within 2–3 days after initial emergence, whereas emergence of other lines (e.g., Golden Cross, Triumph 64 and 90451ARS) was gradual and extended over many days. Eleven lines showed >60% emergence by 21 DAP. The four entries with no emergence at all had coleoptile lengths ranging from 53 to 85 mm (Table 1).

**Table 1:** Coleoptile length (CL), thousand kernel weight (TKW) and emergence percentage of selected cultivars in the world wheat collection having maximum and minimum emergence from deep planting on 15 and 21 days after planting.

Entry Name	CL(mm)	TKW(gm)	% Emergence		
			DAY15	DAY21	
Klein Dragon	51.0	26	52	66	
Fortuna	90.8	33.8	56	64	
Canadian Red	99.1	37.2	58	64	
Luft	82.3	37.2	58	64	
Sonora	106.3	29.8	60	62	
Okla. 61STW8637	60.5	33.3	50	62	
J961051	96.7	33.5	46	62	
Bounty 309	46.8	39.1	4	8	
WA 64250	49.0	32.0	2	4	
Chinook	74.0	39.7	0	0	
Blizzard	85.4	38.7	0	0	
Newton	53.4	39.0	0	0	

**Figure 2:**
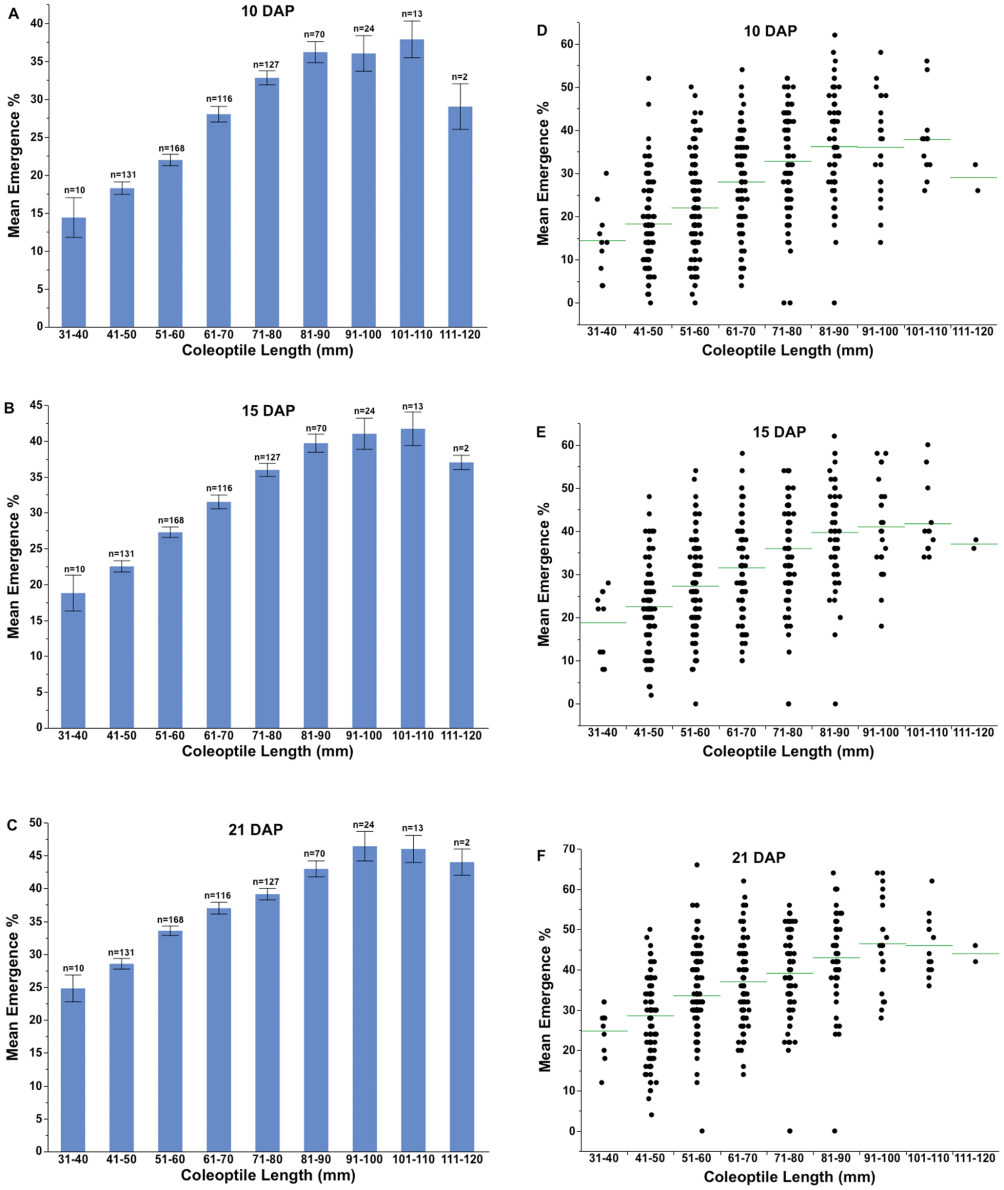
Mean emergence percentage among various coleoptile length classes (A–C) and, range of variation in coleoptile length classes on 10, 15, and 21 days after planting (D–F). The error bar represents the standard error of the mean. Number of lines in each coleoptile length class (n) is given above each bar. The horizontal line represents the mean in each coleoptile class (D–F).

The percent emergence at 10 DAP among coleoptile length classes ranged from 14% to 38%. There was a gradual increase in emergence percentage with increasing coleoptile length up to 90 mm, beyond which there were no statistical differences (Figure 2A). In general, emergence decreased for entries with coleoptile length >110 mm, although this interpretation is based only on two entries. The best early emergers on 7 DAP had coleoptiles ranging from 50–110 mm with no significant improvement in emergence above 60 mm (data not shown). The emergence trend was very similar at 15 and 21 DAP with no significant improvement in emergence of entries with coleoptiles longer than 90 mm (Figure 2B–C). Mean emergence percentage among entries on 10 and 15 DAP ranged from 2 to 62% and on 21 DAP from 4% to 66%. Overall, five of the nine coleoptile length classes differed significantly in their mean percent emergence (Figure 2A–C).

Within each of coleoptile length class, the range of emergence percentage was wide and variable (Figure 2D–F). The entry with the greatest emergence (66%) had a coleoptile length of only 52 mm. The minimum emergence in this (51–60 mm) coleoptile length class was 12%. The emergence percentage of entries with coleoptiles >100 mm (average coleoptile length of 112 mm) was 46% and ranged from 36% to 62%. The within-coleoptile class range was wider at 10 DAP compared to that at 21 DAP, suggesting that final emergence is better correlated with coleoptile length than to emergence speed (Figure 2E–F). Except for the four non-emerging entries that spanned four coleoptile length classes with the highest being 80 to 90 mm, minimum emergence in each coleoptile length class gradually increased with the increasing coleoptile length (Figure 2D–F). The greatest relative emergence improvements occurred for the three shortest coleoptile classes (31–40, 41–50, and 51–60 mm) and then leveled off for the next four classes (Figure 2D–F). Maximum emergence for the two longest coleoptile length classes gradually decreased from 64% in 91–100 mm class to 46% in the 111–120 mm class (Figure 2D–F).

### Correlation Between Emergence and Coleoptile Length

**Figure 3:**
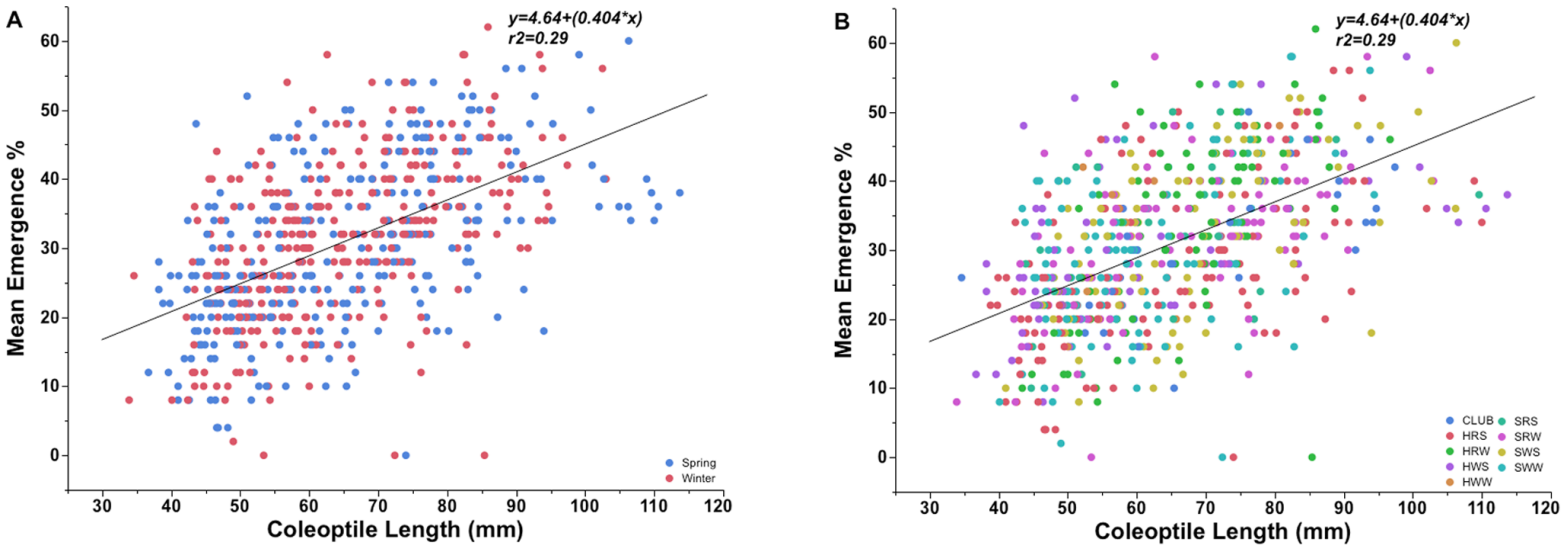
Mean emergence percentage of the (A) spring and winter type and (B) different market class of wheat world collection entries plotted against coleoptile length on 15 days after planting.

There was an overall positive correlation between coleoptile length and emergence from deep planting (Figure 3A–B). On 8 DAP, correlation between emergence and coleoptile length was weak (r^2^ = 0.12, p<0.0001), but somewhat improved on 9 DAP (r^2^ = 0.23, p<0.0001). Coefficients of determination (r^2^) for the relation of coleoptile length with emergence on 10, 15, and 21 DAP were 0.28, 0.29, and 0.23 respectively (p<0.0001) (Figure 3A, data for 10 and 21 DAP not shown).

Coefficients of determination of emergence with coleoptile length were similar among market classes and, except for the HWW class where there was no significant correlation (p = 0.1) on any DAP. Club wheat showed the highest coefficients of determination (p<0.001) on 10, 15 and 21 DAP (r^2^ = 0.43, 0.43, 0.34, respectively).

### Kernel Weight Versus Seedling Emergence

**Figure 4:**
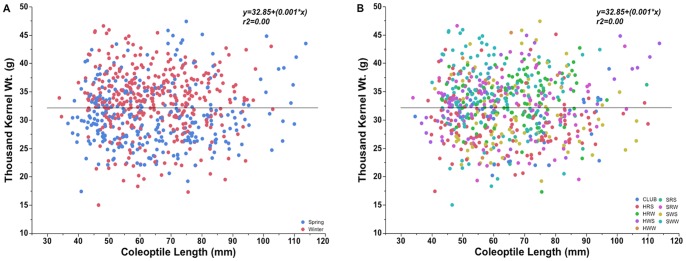
Thousand kernel weight of (A) spring and winter type wheat and (B) different market classes of world wheat collection entries plotted against coleoptile length.

**Figure 5:**
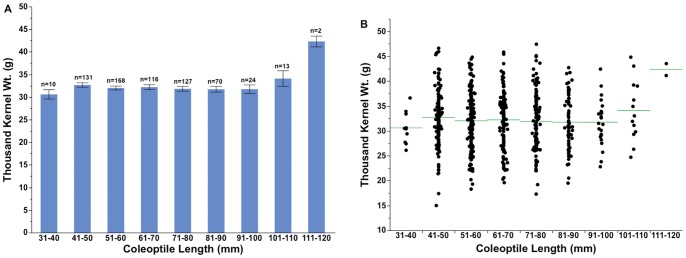
Thousand kernel weight distribution (A) and range variation (B) among different coleoptile length classes. Bars represent the standard error of the mean. The sample size (n) of each coleoptile class length is provided above the data bars. The horizontal line represents the mean in each coleoptile class (B).

Thousand kernel weight (TKW) of the world wheat collection cultivars ranged from 15.0 to 47.4 g and averaged 32.0 g. Mean TKW for winter and spring cultivars was 33.2 and 30.8 g, respectively. There was no overall correlation between coleoptile length and TKW for spring and winter type and all the market classes except for HWS (Figure 4 A–B). The TKW of coleoptile class 31–40 and 110–120 mm entries differed significantly compared to other coleoptile classes (Figure 5A). The widest variability in TKW occurred in coleoptile classes between 41–80 mm (Figure 5B). Although, the coefficient of determination for TKW and seed size was 0.27 (p<0.001), seed size had negligible correlation with coleoptile length (r^2^ = 0.01, p<0.01). The weak correlation held true for both winter and spring types and all market classes except for HWS, which showed the most positive correlation with coleoptile length (r^2^ = 0.22, p<0.001). Thousand kernel weight did not affect emergence on any DAP nor was it associated with plant height at maturity (data not shown).

### Plant Height

**Figure 6:**
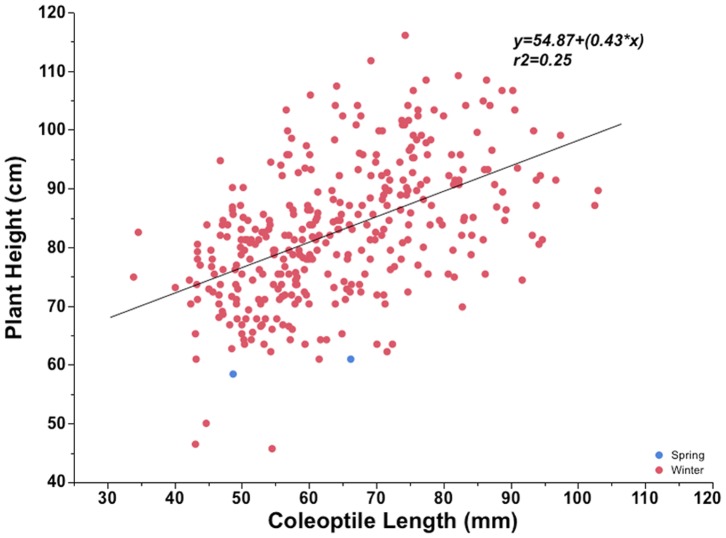
Mean plant height at maturity of spring and winter type cultivars in the world wheat collection entries plotted against coleoptile length.

**Figure 7:**
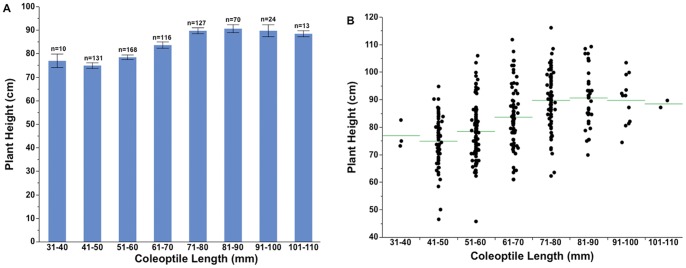
Mean (A) and range variation (B) in plant height at maturity in different coleoptile length classes of the world wheat collection. The bars are the standard error of the mean. The sample size (n) of each coleoptile class is provided above the data bars. The horizontal line represents the mean in each coleoptile class (B).

Plant height at maturity of 357 winter and two spring entries ranged from 46 to 116 cm with an average height of 82 cm. In general, plant height was positively correlated with coleoptile length (r^2^ = 0.25, p<.001, Figure 6). There were no differences in plant height among entries with coleoptiles longer than 80 mm (Figure 7A). The tallest entry had a height of 116 cm with a coleoptile length of 74 mm. The shortest entry had a plant height of 46 cm with a coleoptile length of 54 mm. There was wide variability in plant height within each coleoptile length class (Figure 7B).

## Discussion

Variation among wheat cultivars for coleoptile length and its correlation with seedling emergence has been reported from several authors [13], [29], [30] but the number of cultivars evaluated in those studies was relatively small. Here, we present a comprehensive analysis of variation among 662 wheat cultivars from major wheat growing regions around the world and provide a detailed analysis of the correlation between coleoptile length and seedling emergence.

Our wheat collection was carefully selected to capture the variation present in cultivated wheat while minimizing redundancy. We observed unprecedented variation in the population for the three traits studied. With a range from 34 mm to 114 mm, our collection had more than a three-fold difference in coleoptile lengths. Similarly, variation for seedling emergence from deep planting was high with some lines showing some emergence as early as 7 DAP and four lines with no emergence even on 21 DAP. Variation for seedling emergence was related to speed, extent, and uniformity of final plant stand.

It has previously been reported that the commonly used GA-insensitive semi-dwarf gene mutations (RhtB1b and RhtD1b) reduce coleoptile length by 30–40% compared to standard-height cultivars [15]. The GA insensitivity reduces cell wall extensibility, thus reduces plant height and also negatively affects growth of the coleoptile and first leaf [10], [31]. Coleoptile length variation among the CIMMYT lines in our collection was very narrow with the majority having a coleoptile length between 40–60 mm. This narrow variation is probably due to the semi-dwarf nature of all the CIMMYT lines and/or due to a common genetic background. The CIMMYT lines had an average 32% (ranging from 12–54%) of seedlings emerged on 21 DAP. In addition to genetics, other factors including soil temperature, texture and moisture may also influence coleoptile length [20], [25], [32], [33].

Emergence from deep planting is particularly important in the low-precipitation dryland regions of the world, such as the inland PNW, where wheat is planted as deep as 200 mm with deep-furrow drills and seedlings must emerge through 150 mm or more soil cover. The coleoptile protects the emerging leaf (i.e., until the first leaf protrudes through the tip of the coleoptile while still below the soil surface) and also provides the mechanical support required for seedling emergence. Coleoptile length has previously been reported to be important for seedling emergence. In the present study, coleoptile length was significantly correlated with seedling emergence but accounted for only 28% of the variability for emergence on 10 and 15 DAP (Figure 2A and B). The emergence percentage of entries in coleoptile class 31–40 mm (25%) on 21 DAP was significantly lower than any other class (Figure 3C) with a gradual increase in mean emergence percentage up to a coleoptile length of 90 mm. Comparing various coleoptile length classes, emergence increased more dramatically with increased coleoptile length at 10 DAP compared to that at 21 DAP (Figure 3A and C). Our linear regression coefficients of determination for the relationship of coleoptile length to emergence were considerably lower compared to previous reports [6], [8], [13], [29]. Careful evaluation of present data suggests that coleoptile length is not as critical for seedling emergence as previously thought. Our five best emerging cultivars had coleoptile length range from 51–106 mm. Further, a wide range of emergence was observed within each of the coleoptile length classes. The lower value of emergence decreased with decreasing coleoptile length, suggesting an underlying effect of coleoptile length on emergence, but the maximum emergence values did not increase with coleotiptiles longer than 90 mm, suggesting that coleoptile lengths longer than 90 mm may not be beneficial for emergence.

Several entries in our study with relatively short coleoptiles emerged well from deep planting while other entries with longer coleoptiles had poor emergence. This difference is not due to uneven germination as during the coleoptile length measurement experiment all lines germinated well. Except for the shortest class, each coleoptile length class showed a wide range of seedling emergence, suggesting that coleoptile length is not as strongly correlated to seedling emergence as previously reported, thus making emergence a complex trait. Similar observations were made in barley (*Hordeum vulgare L.*) where lines with widely different coleoptile lengths showed similar seedling emergence [34]. Another reason for range of coleoptile length variation within the coleoptile class might be genotype parentage which affects coleoptile length [35]. For example, club wheat had higher mean coleoptile length among the different market classes studied.

Lower dependence of emergence on coleoptile length in comparison to previous reports may partly be due to our much larger and highly diverse material providing increased precision to study this correlation. Another explanation for the weaker correlation could be that previous studies used wheat lines with limited geographic diversity, whereas our wheat lines collected from around the world may respond differently under different environmental conditions.

In general, seed weight had no effect on coleoptile length or seedling emergence. This is in agreement with most other studies, but there are some reports of a strong correlation between seed weight/size and coleoptile length in wheat [22], [35], [36]. In barley, seed weight had no effect on coleoptile length though it accounted for 31–53% of variability in coleoptile width [37]. In our study, except for the lightest and the heaviest seed weight classes, there were essentially no emergence differences. Coleoptile length and emergence of the lightest seed weight class was significantly lower than the other classes. Similarly, the highest coleoptile length class had significantly higher seed weight compared to the other coleoptile classes. In previous studies reported in the literature, essentially no effect of seed weight was reported on emergence or yield [18], [20], [24]. In our study the diverse genetic nature of the entries in each coleoptile length class might account for the differences in the coleoptile length and emergence within a particular seed weight range.

Coleoptile length showed a positive overall correlation with final plant height in our study (Figure 6). These results are in accordance with the earlier reports [38]. Coleoptile length and plant height are polygenic traits and many QTLs have been reported that influence traits [26], [28], [39]–[41]. We also observed a full range of plant heights in particular coleoptile class e.g. cultivar with short plant height (29 cm) with a long coleoptile (92 mm). As we do not know the nature of dwarfing genes in the world collection, these could possibly be GA sensitive genotypes that are reported to have no adverse effect on coleoptile length but reduce plant height, or possibly have favorable alleles for coleoptile length in semi-dwarf background [41]. In general, plant height was positively correlated with emergence at 10, 15, and 21 DAP. There was a general trend of increased plant height up to 90 mm coleoptile length, but no differences in the mean plant height for entries with coleoptiles longer than 90 mm.

## Conclusions

A collection of 662 carefully selected wheat cultivars from around the world representing a full range of variation for the studied traits allowed us to gain improved understanding of the relation between coleoptile length and emergence from deep planting depths. Refuting the previous claims of coleoptile length explaining 60% or more of the variation for seedling emergence, our extensive study explains only 28% of the variability for seedling emergence related to coleoptile length. Contradictory to earlier reports suggesting a linear relationship between coleoptile length and seedling emergence, our study clearly showed that coleoptiles longer than 90 mm has no positive affect on seedling emergence and may actually have a detrimental effect on emergence from deep planting depths. Further, a full range of emergence percentage within each coleoptile length class clearly suggest the involvement of factors other than coleoptile length in determining the extent as well as speed of seedling emergence from deep planting depths. Results showed that except for the smallest and the largest seed weight classes that evidenced a proportional affect on coleoptile length, a similar affect was not observed for the remaining classes. We observed that the effect of coleoptile length on seedling emergence was different for different wheat market classes. Further, we observed that seed weight has no significant affect on seedling emergence from deep planting. Hence, we conclude that wheat seedling emergence from deep planting depths is a complex trait and further detailed studies are required to understand the underlying mechanisms.

## Supporting Information

Table S1 **Names of the 662 cultivars evaluated in the coleoptile length and emergence experiment.**
(DOCX)
