# Supplementary material for: Wheat Seedling Emergence from Deep Planting Depths and Its Relationship with Coleoptile Length
Source: PLoS One. 2013 Sep 3;8(9):e73314. doi: 10.1371/journal.pone.0073314 (PMC3760894; doi:10.1371/journal.pone.0073314)
Supplement: Table S1 — Names of the 662 cultivars evaluated in the coleoptile length and emergence experiment. (DOCX) [file pone.0073314.s001.docx]

Table S1: Names of the 662 cultivars evaluated in the coleoptile length and emergence experiment.

| 1 | Gabo 60 | 332 | Pavon F 76 |
| --- | --- | --- | --- |
| 2 | Nacozari F 76 | 333 | Sakha 8 |
| 3 | Yecora Rojo 76 | 334 | Chivito |
| 4 | Annapurna 1 | 335 | Hermosillo M77 |
| 5 | CWI50568 | 336 | Seri M 82 |
| 6 | Mexipak65 | 337 | UP262 |
| 7 | Bluebird 15 | 338 | Bahawalpur 79 |
| 8 | Abu Ghraib#3 | 339 | Sakha 69 |
| 9 | Faislabad 83 | 340 | Hartog |
| 10 | Punjab 88 | 341 | Pirsabak 85 |
| 11 | San Cayetano S 97 | 342 | Gonen |
| 12 | BR 18 | 343 | Rayon F 89 |
| 13 | Kenya Kwale | 344 | Nesser |
| 14 | Temporalera M87 | 345 | ICA Yacuanquer |
| 15 | Estanzuela Pelon 90 | 346 | TIA.1 |
| 16 | Cham 6 | 347 | Borlaug M 95 |
| 17 | Tinamouii | 348 | PBW343 |
| 18 | Arivechi M 92 | 349 | Inifap M 97 |
| 19 | Yaqui 50 | 350 | Tobarito M 97 |
| 20 | Narino 59 | 351 | Granero Inta |
| 21 | Penjamo T 62 | 352 | Prointa Oasis |
| 22 | Pitic62 | 353 | Itapua 40-Obligado |
| 23 | Crespo | 354 | Klein Dragon |
| 24 | Nadadores M 63 | 355 | BAW898 |
| 25 | Sonora 64 | 356 | Cumhuriyet 75 |
| 26 | INIA F66 | 357 | Millaleau INIA |
| 27 | Bajio | 358 | IAN 8-Pirapo |
| 28 | Kalyansona | 359 | Pavon |
| 29 | Safed lerma | 360 | Pointa Federal |
| 30 | Sonalika | 361 | Bw21695 |
| 31 | Calidad | 362 | Andes-56 |
| 32 | UP301 | 363 | Sariab-92 |
| 33 | Potam S 70 | 364 | Orofen 60 |
| 34 | Marcos Juarez Inta | 365 | Lerma Rojo 64 |
| 35 | Tanori F 71 | 366 | V-17 |
| 36 | Arz | 367 | PJ62/GB55 |
| 37 | Jupateco F 73 | 368 | Zamindar 80 |
| 38 | Maya 74 | 369 | Pakistan 81 |
| 39 | Salamanca 75 | 370 | Cordillera 3 |
| 40 | Liesbeck | 371 | Idaho 61M3404 |
| 41 | Idaho 62M9-224 | 372 | D 6647 |
| 42 | Lemhi 66 | 373 | ND 467 |
| 43 | 64Ab9405 | 374 | ND 476 |
| 44 | Twin | 375 | Ellar |
| 45 | Owens | 376 | Edmore |
| 46 | IDO190 | 377 | Coteau |
| 47 | IDO232 | 378 | D804 |
| 48 | Copper | 379 | Monroe |
| 49 | Vandal | 380 | D7925 |
| 50 | Idaho 266 | 381 | ND 13-137 |
| 51 | Whitebird | 382 | Amidon |
| 52 | Frex | 383 | Munich |
| 53 | II-53-521 | 384 | Pierce |
| 54 | Chris | 385 | ND 2710 |
| 55 | II-55-1 | 386 | STW 598874 |
| 56 | II-58-60 | 387 | YSCA-1 |
| 57 | II-62-78 | 388 | Sel. 90 |
| 58 | MN 6616M | 389 | WA 6101 |
| 59 | Wheaton | 390 | WA 7175 |
| 60 | II-64-20 | 391 | Spillman |
| 61 | MN 6898 | 392 | ARS95 451 |
| 62 | Vance | 393 | ARS95 457 |
| 63 | Norm | 394 | Eden |
| 64 | Verde | 395 | Alpowa |
| 65 | McVEY | 396 | Alturas |
| 66 | Justin | 397 | Challis |
| 67 | ND 202-2 | 398 | Edwall |
| 68 | ND 271 | 399 | Penawawa |
| 69 | ND 229-1 | 400 | Jubilee |
| 70 | ND 287 | 401 | Vanna |
| 71 | Fortuna | 402 | Tara 2002 |
| 72 | Leeds | 403 | Scarlet |
| 73 | ND 59-120A | 404 | Jefferson |
| 74 | ND 407 | 405 | Hollis |
| 75 | Waldron | 406 | Calorwa |
| 76 | ND 22 | 407 | Zak |
| 77 | ND 66 | 408 | Wawawai |
| 78 | CI014952 | 409 | Centennial |
| 79 | CI014953 | 410 | Macon |
| 80 | Rolette | 411 | Lolo |
| 81 | Klasic | 412 | Bounty 208 |
| 82 | IDO377s | 413 | Moran |
| 83 | Yecora Rojo | 414 | Union |
| 84 | Saxon | 415 | Utac |
| 85 | Newana | 416 | White Fife |
| 86 | Urquie | 417 | White Marquis |
| 87 | Rushmore | 418 | Sea Island |
| 88 | Ramona | 419 | Ruby |
| 89 | Hard Federation | 420 | Rival |
| 90 | Redchaff | 421 | Lemhi |
| 91 | Selkirk | 422 | Little Club |
| 92 | Centana | 423 | Marfed |
| 93 | Redman | 424 | Touse |
| 94 | Saunders | 425 | Thatcher |
| 95 | Lee | 426 | Supreme |
| 96 | Peak | 427 | Spinkcota |
| 97 | Probrand 751 | 428 | Sonora |
| 98 | Wadual | 429 | Galgalos |
| 99 | Wakanz | 430 | Federation 67 |
| 100 | Canthatch | 431 | Federation |
| 101 | Conley | 432 | Reward |
| 102 | Peak 72 | 433 | Rescue |
| 103 | Prospur | 434 | Reliance |
| 104 | Kitt | 435 | Regent |
| 105 | Wampum | 436 | Red Bobs |
| 106 | Walladay | 437 | Ramona 50 |
| 107 | Pondera | 438 | Orfed |
| 108 | Sterling | 439 | Oregon Zimmerman |
| 109 | McKay | 440 | Onas 53 |
| 110 | Waid | 441 | Onas |
| 111 | Irridur | 442 | Mida |
| 112 | Norana | 443 | Marquis |
| 113 | Olaf | 444 | Pacific Bluestem |
| 114 | Borah | 445 | Pacific Bluestem 37 |
| 115 | Waverly | 446 | Pilot |
| 116 | Treasure | 447 | Premier |
| 117 | Westbred 906R | 448 | Allen |
| 118 | Westbred 911 | 449 | Awned Onas |
| 119 | Bliss | 450 | Baart Early Selection |
| 120 | Ward | 451 | Canadian Red |
| 121 | Cadet | 452 | Era |
| 122 | Bluechaff | 453 | Bounty 309 |
| 123 | Big Club | 454 | Winsome |
| 124 | Hard Federation-31 | 455 | Aim |
| 125 | Henry | 456 | Bronze Chief |
| 126 | Hope | 457 | Kodiak Dwarf |
| 127 | Hybrid 63 | 458 | Kubanka |
| 128 | Hybrid 143 | 459 | Kahla |
| 129 | Kinney | 460 | Sentry |
| 130 | Kenhi | 461 | Langdon |
| 131 | Ceres | 462 | Wells |
| 132 | Westbred Express | 463 | Wandell |
| 133 | Lagoda | 464 | Produra |
| 134 | Flomar | 465 | WL 444 |
| 135 | Hybrid 123 | 466 | Pomerelle |
| 136 | Dicklow | 467 | Florence |
| 137 | Gypsum | 468 | Buchanan |
| 138 | Hyper | 469 | ORCF-101 |
| 139 | Idaed | 470 | Weatherford |
| 140 | Indian | 471 | Tubbs |
| 141 | Baart 46 | 472 | Mohler |
| 142 | New Zealand | 473 | MJ9 |
| 143 | Beaver | 474 | MJ4 |
| 144 | Pilcraw | 475 | Finch |
| 145 | Rink | 476 | Hubbard |
| 146 | Surprise | 477 | MacVicar |
| 147 | White Federation | 478 | Albion |
| 148 | Bunyip | 479 | Brundage 96 |
| 149 | Currawa | 480 | NuFrontier |
| 150 | Wilbur | 481 | NuHorizon |
| 151 | Early Baart | 482 | W96-054 |
| 152 | Major | 483 | Paladin |
| 153 | Lemhi 53 | 484 | Finley |
| 154 | Springfield | 485 | Hatton |
| 155 | Fielder | 486 | Moreland |
| 156 | Fieldwin | 487 | Quantum-HYB-542 |
| 157 | Schlanstedt | 488 | Residence |
| 158 | Preston | 489 | Semper |
| 159 | Chinook | 490 | Symphony |
| 160 | Manitou | 491 | Blizzard |
| 161 | Red River 68 | 492 | Rohde |
| 162 | Temple | 493 | Triumph |
| 163 | Foote | 494 | Turkey |
| 164 | Nuhills | 495 | Wasatch |
| 165 | Chukar | 496 | Westmont |
| 166 | Bruehl | 497 | Schlanstedts Sommerweizen |
| 167 | Moro | 498 | Ruddy |
| 168 | Edwin | 499 | Rio |
| 169 | OR2010010 | 500 | Ridit |
| 170 | Rew | 501 | Wilhelmina |
| 171 | McDermid | 502 | Yogo |
| 172 | Luke | 503 | Lofthouse |
| 173 | Hyslop | 504 | Tendoy |
| 174 | Yamhill | 505 | Gold Drop |
| 175 | Luft | 506 | Golden |
| 176 | Genesee Giant | 507 | Genro |
| 177 | Rex M2 | 508 | Fultz |
| 178 | PI558510 | 509 | Elmar |
| 179 | Lambert | 510 | Elgin |
| 180 | Hiller | 511 | Early Blackhull |
| 181 | Mediterranean | 512 | Requa |
| 182 | Norin 10/ Brevor 14 | 513 | Red Russian |
| 183 | Barbee | 514 | Omar |
| 184 | Stephens | 515 | McCall |
| 185 | Greer | 516 | Oro |
| 186 | Jacmar | 517 | Early Genesee Giant |
| 187 | Lenore | 518 | Huston |
| 188 | Crew | 519 | Pride of Genesee |
| 189 | Gene | 520 | Purplestraw |
| 190 | Syringa | 521 | Alba |
| 191 | Malcom | 522 | Alicel |
| 192 | Madsen | 523 | Arco |
| 193 | Hyak | 524 | Athena |
| 194 | Oveson | 525 | Brevor |
| 195 | John | 526 | Blue Jacket |
| 196 | Cashup | 527 | Blackhull |
| 197 | Basin | 528 | Bison |
| 198 | ID 571 | 529 | Golden Cross |
| 199 | Hill 81 | 530 | Harvest Queen |
| 200 | PI178383 | 531 | Hood |
| 201 | Kiowa | 532 | Dale |
| 202 | Coulee | 533 | Hymar |
| 203 | Ark | 534 | Survivor |
| 204 | Crest | 535 | Idaho 60BF4 |
| 205 | Wanser | 536 | Idaho 9129 |
| 206 | Ute | 537 | ID 701005 |
| 207 | Relief | 538 | ID 701008 |
| 208 | Ranger | 539 | Weston |
| 209 | Dual | 540 | ID 74-5101-30 |
| 210 | Neeley | 541 | IDO022 |
| 211 | Cheyenne | 542 | Idaho 352 |
| 212 | Chiefkan | 543 | Idaho 364 |
| 213 | Columbia | 544 | IDO562 |
| 214 | White Winter | 545 | Purdue 5157-10 |
| 215 | Dawson | 546 | Knox 62 |
| 216 | Goldcoin | 547 | Purdue WW23 |
| 217 | Hybrid 128 | 548 | Purdue WW26 |
| 218 | Albit | 549 | Riley 67 |
| 219 | Powerclub | 550 | Vel |
| 220 | Rex | 551 | Oasis |
| 221 | Druchamp | 552 | Key |
| 222 | Peck | 553 | Fuzz |
| 223 | Faro | 554 | Beau |
| 224 | Raeder | 555 | Downy |
| 225 | Dusty | 556 | Sullivan |
| 226 | Jones Fife | 557 | Purdue TBR 26-6-4 |
| 227 | Coppei | 558 | Elmo |
| 228 | Sun (Sol) | 559 | Compton |
| 229 | Triplet | 560 | Clarafay |
| 230 | Thorne | 561 | P 5714B3-11-3-1-1 |
| 231 | Spokane Chief | 562 | Steele |
| 232 | Montana 36 | 563 | Patterson |
| 233 | Tenmarq | 564 | PR143 |
| 234 | Cache | 565 | PR302 |
| 235 | Itana | 566 | Goldfield |
| 236 | Delmar | 567 | II-51-6 |
| 237 | Bridger | 568 | II-53-72 |
| 238 | Franklin | 569 | Cornell 54117aB-2B-5 |
| 239 | Hansel | 570 | NY 5660bB-3BW-3 |
| 240 | Heglar | 571 | NY 5726aB-3B-11 |
| 241 | Jeff | 572 | Yorkstar |
| 242 | Arbon | 573 | Ticonderoga |
| 243 | Winridge | 574 | Houser |
| 244 | 1480844 | 575 | TXGBE272 |
| 245 | 66152-4 | 576 | TXGBE307 |
| 246 | NY 6432-18 | 577 | TX91D7012 |
| 247 | Geneva | 578 | TAM 111 |
| 248 | NY batavia | 579 | Gaines |
| 249 | Agassiz | 580 | Sel. 1 |
| 250 | Triumph 64 | 581 | Sel. 7 |
| 251 | Okla. 61STW8617 | 582 | Nugaines |
| 252 | Okla. 61STW8637 | 583 | Omar Mutant 448A |
| 253 | Nicoma | 584 | Paha |
| 254 | OK 627530 | 585 | WA 64250 |
| 255 | STW 646340 | 586 | WA 6415606 |
| 256 | STW 646408 | 587 | WA 6415619 |
| 257 | STW 646378 | 588 | WA 669897 |
| 258 | Osage | 589 | Sprague |
| 259 | Rall | 590 | WA 5911 |
| 260 | OK 78321 | 591 | Daws |
| 261 | OK 78R7228 | 592 | WA 5841 |
| 262 | OK 79R6863 | 593 | WA 5835 |
| 263 | OK 79256 | 594 | REA 77-2 |
| 264 | Chisholm | 595 | Lewjain |
| 265 | Century | 596 | 79-3 |
| 266 | OK66R5803 | 597 | 79-10 |
| 267 | OK 75R3645 | 598 | VH078265 |
| 268 | OK 75R3741 | 599 | WA 7117716 |
| 269 | OK 77R6699 | 600 | Rely |
| 270 | OK91G107 | 601 | 90451ARS |
| 271 | OK92G201 | 602 | 90457ARS |
| 272 | OK92G206 | 603 | WA 7665 |
| 273 | OK91P648 | 604 | WA 7625 |
| 274 | TX 333-56-18 | 605 | WA 7624 |
| 275 | TX 391-56-D1-1 | 606 | Coda |
| 276 | TX 1826-1 | 607 | WA7690 |
| 277 | TX 60C4968 | 608 | WA7770 |
| 278 | TX 60C4965 | 609 | ARS95 452 |
| 279 | TAM 105 | 610 | ARS95 454 |
| 280 | TAM 107 | 611 | ARS95 460 |
| 281 | TX 78V2408 | 612 | Kansas No. 5664 |
| 282 | TX 76-40-2 | 613 | Kansas No. 1214-5 |
| 283 | TAM 109 | 614 | Rodco |
| 284 | TX 85-264 | 615 | Kansas No. 6322 |
| 285 | Kansas No. 56655 | 616 | NE 80413 |
| 286 | Kansas No. 62337 | 617 | NE 82652 |
| 287 | Chanute | 618 | Arapahoe |
| 288 | Kansas No. 594-2 | 619 | NE 73868 |
| 289 | Kirwin | 620 | Pronghorn |
| 290 | Parker 76 | 621 | NE91651 |
| 291 | Newton | 622 | 94L10524 |
| 292 | KS78H9233 | 623 | Millennium |
| 293 | KS82H1640HF | 624 | NW97S343 |
| 294 | Norkan | 625 | 97L9521 |
| 295 | KS 70356 | 626 | Harry |
| 296 | MW10 | 627 | N02Y4514 |
| 297 | Karl | 628 | N98L20040-44 |
| 298 | KS87UP9 | 629 | ARS 98237 |
| 299 | Arlin | 630 | Bauermeister |
| 300 | KS91WGRC11 | 631 | Boundary |
| 301 | KS92WGRC15 | 632 | Columbia-1 |
| 302 | KS92WGRC26 | 633 | Eltan |
| 303 | Colby 94 | 634 | Golden Spike |
| 304 | HBC696-108 | 635 | GT123-64 |
| 305 | KS92WGRC20 | 636 | J961051 |
| 306 | Jagger | 637 | WA7916 |
| 307 | KS96WGRC40 | 638 | WA7917 |
| 308 | LG512S | 639 | Batum |
| 309 | Nebr. Sel. 533570 | 640 | Tyee |
| 310 | Nebr. Sel. 422121 | 641 | WPB470 |
| 311 | Scout | 642 | WA7690 SEL |
| 312 | Nebr. Sel. 524878 | 643 | Norstar |
| 313 | LG518S | 644 | Sadash |
| 314 | Nebr. Sel. 61904 | 645 | Bhishaj |
| 315 | NB 62378 | 646 | Andrew |
| 316 | NB 65312 | 647 | SWS366 |
| 317 | Trapper | 648 | WA008058 |
| 318 | NB 67730 | 649 | WA008059 |
| 319 | NB 66548 | 650 | Eunupa |
| 320 | Centurk | 651 | Woori |
| 321 | NB 66461 | 652 | Olgueru |
| 322 | Lancota | 653 | Rod |
| 323 | Siouxland | 654 | Mac1/Ptarmigan |
| 324 | Cody | 655 | MDM |
| 325 | NE 78702 | 656 | Xerpha |
| 326 | Guide | 657 | Simon |
| 327 | PI511673 | 658 | ORCF-101 |
| 328 | Betty | 659 | ORCF-102 |
| 329 | Cara | 660 | LG510S |
| 330 | Masami | 661 | Ptarmigan/Augusta//Brundage96 |
| 331 | Tubbs 06 | 662 | Brundage96/WPB470 |
